# Evaluation of the intra- and inter-specific genetic variability of *Plasmodium *lactate dehydrogenase

**DOI:** 10.1186/1475-2875-6-140

**Published:** 2007-10-25

**Authors:** Arthur M Talman, Linda Duval, Eric Legrand, Véronique Hubert, Seiha Yen, David Bell, Jacques Le Bras, Frédéric Ariey, Sandrine Houze

**Affiliations:** 1 Unité d'Epidémiologie Moléculaire, Institut Pasteur in Cambodia 5 Boulevard Monivong BP983, Phnom Penh, Kingdom of Cambodia; 2 Division of Cell and Molecular Biology, Imperial College London, SW72AZ London, UK; 3 Centre National de Référence sur la Chimiorésistance du Paludisme dans la région Antilles-Guyane, Institut Pasteur de la Guyane, BP 6010, F-97306 Cayenne-Cedex, France; 4 Centre National de Référence du Paludisme, AP-HP, Hôpital Bichat-Claude Bernard, Paris, France; 5 Malaria, and other Vector-borne and Parasitic Diseases, World Health Organization-Regional Office for the Western Pacific, PO Box 2932, Manila, Philippines

## Abstract

**Background:**

Malaria diagnosis is vital to efficient control programmes and the recent advent of malaria rapid diagnostic tests (RDTs) provides a reliable and simple diagnostic method. However a characterization of the efficiency of these tests and the proteins they detect is needed to maximize RDT sensitivity.

**Methods:**

Plasmodial lactate dehydrogenase (*pLDH*) gene of wild isolates of the four human species of *Plasmodium *from a variety of malaria endemic settings were sequenced and analysed.

**Results:**

No variation in nucleotide was found within *Plasmodium falciparum*, synonymous mutations were found for *Plasmodium malariae *and *Plasmodium. vivax*; and three different types of amino acid sequence were found for *Plasmodium ovale*. Conserved and variable regions were identified within each species.

**Conclusion:**

The results indicate that antigen variability is unlikely to explain variability in performance of RDTs detecting pLDH from cases of *P. falciparum, P. vivax *or *P. malariae *malaria, but may contribute to poor detection of *P. ovale*.

## Background

Rapid and reliable diagnosis is one of the key factors in promoting malaria control. The gold standard for malaria diagnosis remains the examination of Giemsa-stained smears by light microscopy. Whilst this standard has a good sensitivity and specificity and allows species and stage differentiation, it does require the expertise of a trained and experienced microscopist, is time-consuming (30 minutes per diagnostic) and requires equipment not always available or maintainable in remote areas. The 1990's have seen the advent of a new rapid diagnostic method, the immunochromatography-based malaria Rapid Diagnostic Tests (RDTs). These assays are fast (revealed in 15 minutes) and, for the most part, very simple to use. Moreover with the change of therapeutic practice towards relatively expensive artemisinin-based combination therapies [1], a good diagnostic has become essential to limit inappropriate treatment and the development of resistance. Although the use of RDTs has spread, their reliability is still questioned in numerous studies [2,3]. These assays detect one or several antigens, the most common are: histidine-rich protein-2 (HRP-2), aldolase and lactate dehydrogenase (pLDH).

Lactate dehydrogenase is an enzyme that catalyzes the inter-conversion of lactate into pyruvate. This enzyme is essential for energy production in *Plasmodium *[4]. The level of pLDH in the blood has been directly linked to the level of parasitaemia [5].

The genetic diversity of HRP2 has been examined and partly linked to RDT detection sensitivity [6], the genetic variability has also been assessed in aldolase, it has been ruled out as a possible cue for variation RDT sensitivity [7]. Here is the first study of *Plasmodium *LDH genetic variability as a possible cause of variation in sensitivity of RDTs.

## Methods

**Figure 1:**
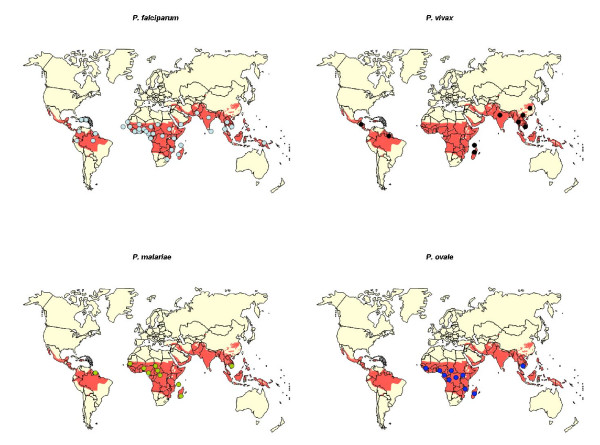
Worldwide distribution of the isolate sequenced in the study, grouped by species. One dot corresponds to one isolate, in red the malaria endemic area.

A total of eight *Plasmodium *species (*Plasmodium falciparum, Plasmodium vivax, Plasmodium. ovale, Plasmodium malariae, Plasmodium yoeli, Plasmodium chabaudi, Plasmodium berghei and Plasmodium. reichnowi*), including the four human pathogens, from numerous origins (Figure 1) were examined with a nested-PCR assay amplifying a 543 bp fragment: corresponding to the 57 to 237 amino acid position of the reference *P. falciparum *LDH coding sequence (pf13_0141). All field samples analysed were diagnosed by microscopic examination and confirmed by PCR [8] and conserved from previous studies and approved at the time by respective National Ethics Committees. Two sets of PCR and nested primers were designed for this study based on the sequences available on GenBank (Table 1) one set use for *P. vivax *and *P. falciparum*, and the other for *P. ovale *and *P. malariae*.

**Table 1:** PCR and nested-PCR primers used in the study

PCR primers	Primer sequence 5' to 3'
Fv1	ATGATYGGAGGMGTWATGGC
Fv2	GCCTTCATYCTYTTMGTYTC
Mo1	ATGATWGGAGGTGTTATGGC
Mo2	TGTGTCCRTATTGDCCTTC
Nested Primers	
Fv1n	AATGTKATGGCWTATTCMAATTGC
Fv2n	AACRASAGGWGTACCACC
Mo1n	TAGGMGATGTTGTTATGTTYG
Mo2n	ATTTCRATAATAGCAGCAGC

Forty PCR cycle were undertaken using 94°C for 30 s, 55°C for 60 s and 72°C for 75 s; the same cycle was used for the nested-PCR but only repeated 35 times. Positive and negative controls were included in all amplification assays. The amplified products were purified using a Quiaquick PCR purification kit (QIAGEN, Valencia, CA) according to the manufacturer's recommendations, and sequenced using Big Dye Terminator kit v1.1 (Applied Biosystems, Foster City, CA) in an AbiPrism 3130 sequencing machine (Applied Biosystems, Foster City, CA).

## Results

No variability was observed in *P. falciparum *(n = 49) with a homology of 100% between all newly sequenced sequences (named F). A single reference sequence on GenBank (corresponding to the FCC1/HN strain) exhibited a different amino acid sequence (named F1). For *P. vivax *(n = 10), four different types of sequence were found, the mutations observed were all synonymous (named V); no geographic pattern was identified. *P. malariae *(n = 17) exhibited three different type of sequences, one for African and American isolates and the other two for the south-east Asian isolate and reference strain respectively. Those variations resulted in the same amino acid sequence (named M).

*P. ovale *(n = 13) exhibited three different types of nucleotide sequences, leading to three different types of amino acid sequences (named O1, O2 and O3). *P. berghei *and *P. yoeli *sequences exhibited synonymous mutations (named Y). *P. chabaudi *exhibited a nucleotide sequence (named C).*P. reichnowi *and *P. falciparum *sequences exhibited synonymous mutations.

**Figure 2:**
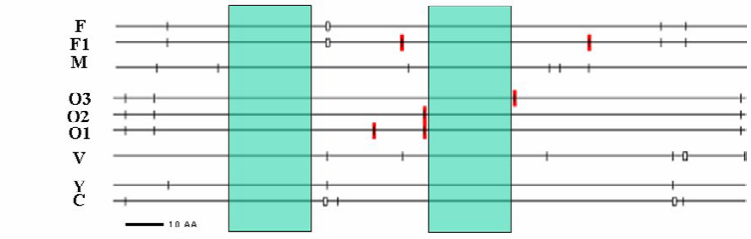
Schematic representation of the 181 amino acid sequence variation (each different marks correspond to one amino acid change). F, F1, M, V, O1, O2, O, Y and C correspond to the sequence identified in the study (see result). The conserved regions of the *Plasmodium *pLDH gene for all species are highlighted in green.

Interestingly a comparison of the sequences of different species reveals the existence of conserved regions and other very variable ones; this inter-specific variation is exhibited in Figure 2. Table 2 gives details of the analysed isolates.

**Table 2:** Result of the sequence analysis for the isolates tested in this paper.

ID Code	Year	species	Origin	Seq	AA
5353A	2005	PF	South Africa	F	F
5353B	2005	PF	South Africa	F	F
5421	2005	PF	Benin	F	F
5445	2005	PF	Brazil	F	F
4899	2004	PF	Burkina Faso	F	F
CAMBF	2001	PF	Cambodia	F	F
5203	2005	PF	Cameroon	F	F
5848	2005	PF	Cap Verde	F	F
5265	2005	PF	Republic of Central Africa	F	F
3414	2002	PF	Colombia	F	F
4682	2004	PF	Comoros	F	F
5405	2005	PF	Congo	F	F
4919	2004	PF	Ivory Cost	F	F
5600	2005	PF	Dominican Republic	F	F
1628	1999	PF	Ecuador	F	F
5648	2005	PF	Gabon	F	F
5083	2004	PF	Gambia	F	F
5094	2004	PF	Ghana	F	F
5898	2005	PF	Guinea	F	F
5339	2005	PF	Equatorial Guinea	F	F
FguyF	2003	PF	French Guiana	F	F
5555	2005	PF	Haiti	F	F
5745	2005	PF	India	F	F
2038	2000	PF	Kenya	F	F
4548	2004	PF	Liberia	F	F
4609	2004	PF	Madagascar	F	F
2686	2001	PF	Malaysia	F	F
5296	2005	PF	Malawi	F	F
5173	2004	PF	Mali	F	F
5793	2005	PF	Mali	F	F
4807	2004	PF	Mauritania	F	F
4629	2004	PF	Mozambique	F	F
5323	2005	PF	Namibia	F	F
5822	2005	PF	Niger	F	F
4582	2004	PF	Nigeria	F	F
5846	2005	PF	Pakistan	F	F
1317	1998	PF	Papua New Guinea	F	F
5225	2005	PF	Sao Tome	F	F
4838A	2004	PF	Senegal	F	F
4512	2004	PF	Sierra Leone	F	F
4764	2004	PF	Sir Lanka	F	F
4562	2004	PF	Sudan	F	F
5224	2005	PF	Tanzania	F	F
5647	2005	PF	Chad	F	F
604	1997	PF	Thailand	F	F
4751A	2004	PF	Togo	F	F
4751B	2004	PF	Togo	F	F
542	1997	PF	Yemen	F	F
5197	2005	PF	Congo Democratic Republic	F	F
ID Code	Year	species	Origin	Seq	AA
Plasmodium malariae					
CAMBM	2001	PM	Cambodia	M2	M
3413	2002	PM	Cameroon	M3	M
4739	2004	PM	Cameroon	M3	M
5990	2006	PM	Cameroon	M3	M
1909	1999	PM	Republic of Central Africa	M3	M
3670	2002	PM	Comoros	M3	M
4014	2003	PM	Comoros	M3	M
1548	1999	PM	Congo	M3	M
2667	2001	PM	Ivory Cost	M3	M
5041	2004	PM	Ivory Cost	M3	M
4568	2004	PM	French Guiana	M3	M
4774	2004	PM	Madagascar	M3	M
516	1997	PM	Senegal	M3	M
1018	1998	PM	Togo	M3	M
2389	2000	PM	Congo Democratic Republic	M3	M
Plasmodium ovale					
5894	2005	PO	Angola	O2	O2
CAMBO	2001	PO	Cambodia	O2	O2
3044	2001	PO	Republic of Central Africa	O2	O2
5979	2006	PO	Ivory Cost	O2	O2
3149	2002	PO	Gabon	O2	O2
4646	2004	PO	Guinea	O2	O2
3740	2002	PO	Congo Democratic Republic	O2	O2
4419	2003	PO	Cameroon	O3	O3
5401	2005	PO	Madagascar	O3	O3
2132	2000	PO	Mali	O3	O3
5994	2006	PO	Mali	O3	O3
2668	2001	PO	Rwanda	O3	O3
3043	2001	PO	Zimbabwe	O3	O3
Plasmodium vivax					
3019	2001	PV	French Guiana	V1	V
1977	2000	PV	India	V1	V
1866	1999	PV	Nicaragua	V1	V
800	1997	PV	Thailand	V1	V
2642	2001	PV	Madagascar	V2	V
5315	2005	PV	Chine	V3	V
CAMBV	2001	PV	Cambodia	V4	V
5753	2005	PV	Comoros	V4	V
1173	1998	PV	Laos	V4	V
					
ID Code		species	Origin	Seq	AA
Reference strains					
3D7		PF	pf13_0141	F	F
FCC1/HN		PF	dq825436	F1	F
EMBL		PM	ay486059	M1	M
EMBL		PO	ay486058	O1	O1
EMBL		PV	ay486060	V1	V
					
YOELII		PY	xm_719008	Y	Y
CHABAUDI		PC	xm_740087	C	C
BERGHEI		PB	ay437808	B	Y
REICHNOWII		PR	ab122147	R	F

Seq = Nucleotide sequence, AA = aminoacid sequence

## Discussion

Here is described, for the first time, the sequence variability of pLDH in the four human's species of malaria and four animal *Plasmodium *species and analysed them together with published sequences. The results indicate the existence of both variable and conserved regions in plasmodial lactate dehydrogenase.

The intra-specific geographic conservation of pLDH suggests that genetic variability may not be linked to disparities in sensitivities or specificities observed in the detection of *P. falciparum *[3] with anti-pan LDH antibodies. The *falciparum*-specific epitope detected by RDTs is probably situated in the inter-specific variable regions we have identified; whilst the pan-malarial epitope is more likely situated in a conserved region. However, Moody *et al*. [2] reported that one pan-specific monoclonal antibody used in a pLDH RDT has a lower affinity to *P. malariae *and *P. ovale *antigens, the attribution of this to a sequence divergence must not be neglected and should be further investigated.

## Conclusion

The WHO states: "Rapid diagnostic tests (RDTs) offer the potential to provide accurate diagnosis to all at risk populations (...) The success of RDTs in malaria control will depend on good quality planning and implementation" [9]. Moreover a rapid diagnostic test needs to be reliable globally, to detect an antigen that mirrors accurately blood parasitaemia; therefore part of a good quality assurance is to monitor such factors.

As part of this quality assurance, we have identified that an intra-specific genetic variability is not a significant factor in the variation of efficiency observed in rapid diagnostic tests in the detection of *P. falciparum*, *P vivax *and *P. malariae*, although it may explain the poor sensitivity to *P. ovale *[7]. Similar findings of low variability have been demonstrated for plasmodial aldolase another target antigen of MRDTs [10] despite a bad sensitivity in the dectection of *P. ovale *infection [11] in contrast to HRP2, a target antigen of *P. falciparum *with high variability affecting MRDT sensitivity. In this regard, pLDH offers advantages as a target antigen for diagnosis. The identification of pan-specific and species-specific regions may help in development of more sensitive and specific monoclonal antibodies for MRDTs.

## Authors' contributions

FA, DB, JLB and SH designed the study and contribute to the discussion. SH, JLB, EL and FA provide specimens for sequencing. SY, AMT, EL, VH and LD process samples and analysed the data. AMT write the first draft of the manuscript, then EL, SH, JLB, FA, DB critically reviewed the manuscript. All authors read and approved the final manuscript.
